# Cardiovascular magnetic resonance in heart transplant patients: diagnostic value of quantitative tissue markers: T2 mapping and extracellular volume fraction, for acute rejection diagnosis

**DOI:** 10.1186/s12968-018-0480-9

**Published:** 2018-08-27

**Authors:** Emmanuelle Vermes, Clémence Pantaléon, Adrien Auvet, Nicolas Cazeneuve, Marie Christine Machet, Anne Delhommais, Thierry Bourguignon, Michel Aupart, Laurent Brunereau

**Affiliations:** 1 Department of Cardiothoracic Surgery, University François Rabelais, Tours, France; 2 Department of Radiology, University François Rabelais, Tours, France; 3 Department of Anatomopathology, University François Rabelais, Tours, France

**Keywords:** Acute cardiac rejection, Endomyocardial biopsy, Cardiovascular magnetic resonance, T1 and T2 mapping

## Abstract

**Background:**

The diagnosis of acute rejection in cardiac transplant recipients requires invasive technique with endomyocardial biopsy (EMB) which has risks and limitations. Cardiovascular magnetic resonance imaging (CMR) with T2 and T1 mapping is a promising technique for characterizing myocardial tissue. The purpose of the study was to evaluate T2, T1 and extracellular volume fraction (ECV) quantification as novel tissue markers to diagnose acute rejection.

**Methods:**

CMR was prospectively performed in 20 heart transplant patients providing 31 comparisons EMB-CMR. CMR was performed close to EMB. Images were acquired on a 1.5 Tesla scanner including T2 mapping (T2 prepared balanced steady state free precession) and T1 mapping (modified Look-Locker inversion recovery sequences: MOLLI) at basal, mid and apical level in short axis view. Global and segmental T2 and T1 values were measured before and 15 min (for T1 mapping) after contrast administration.

**Results:**

Acute rejection was diagnosed in seven patients: six cellular rejections (4 grade IR, 2 grade 2R) and one antibody mediated rejection. Patients with acute rejection had significantly higher global T2 values at 3 levels: 58.5 ms [55.0–60.3] vs 51.3 ms [49.5–55.2] (*p* = 0.007) at basal; 55.7 ms [54.0–59.7] vs 51.8 ms [50.1–53.6] (*p* = 0.002) at median and 58.2 ms [54.0–63.7] vs 53.6 ms [50.8–57.4] (*p* = 0.026) at apical level. The area under the curve (AUC) for each level was 0.83, 0.79 and 0.78 respectively. Patients with acute rejection had significantly higher ECV at basal level: 34.2% [32.8–37.4] vs 27.4% [24.6–30.6] (*p* = 0.006). The AUC for basal level was 0.84. The sensitivity, specificity and diagnosis accuracy for basal T2 (cut off: 57.7 ms) were 71, 96 and 90% respectively; and for basal ECV: (cut off 32%) were 86, 85 and 85% respectively. Combining basal T2 and basal ECV allowed diagnosing all acute rejection and avoiding 63% of EMB.

**Conclusions:**

In heart transplant patients, a combined CMR approach using T2 mapping and ECV quantification provides a high diagnostic accuracy for acute rejection diagnosis and could potentially decrease the number of routine EMB.

## Background

Heart transplantation is the therapy of choice for end-stage heart failure to improve survival and quality of life. Survival after cardiac transplantation is linked to the occurrence of complications, especially the risk of acute rejection during the first year [1, 2]. Approximatively 20 to 40% of patients have at least one episode of acute rejection during the first year [3]. Clinical monitoring and echocardiography have failed to detect early rejection [4]. So far, endomyocardial biopsy (EMB) is the gold standard procedure to diagnose acute rejection. Although there is no international consensus on the optimal frequency of EMB, an average of 10 to 15 biopsies are performed during the first year [5]. The procedure requires radioscopy (irradiation) and a large introduction system. This is an invasive procedure with 0.5 to 3% of complications [6, 7]. Most serious complications are tamponade by cardiac perforation, ventricular arrhythmia and tricuspid valve injury leading to regurgitation [8]. Moreover, the accuracy of EMB to diagnose rejection is imperfect due to a non-uniform pathologic process [9]. Indeed, biopsy negative rejection (true rejection with negative biopsy) is a well-known recognized entity and represents 10 to 20% of rejection [10]. There is also significant interobserver variability in the histologic interpretation of myocardial samples [11]. Clinicians need a reliable and non-invasive process for diagnosing rejection and for limiting the number of biopsies performed.

Rejection in heart transplantation is mediated either by T lymphocytes that have been activated against donor antigens (cellular rejection) or by activated B cells secreting antibody molecules (antibody-mediated rejection: AMR). These processes lead to cellular infiltration, myocardial oedema and inflammation with eventual myocytes damage and haemorrhage [12].

Cardiovascular magnetic resonance (CMR) is the gold standard imaging modality for assessing cardiac morphology, ventricular volumes, systolic function and myocardial mass [13]. In addition, CMR allows for assessing the activity of inflammatory changes using markers for myocardial oedema, hyperaemia, capillary leak and irreversible injury applying a combination of non-contrast (T2 weighted imaging and more recently parametric mapping techniques (T1 and T2 mapping) and gadolinium enhanced technique [14–16].

Native T1 values are higher with increased extracellular compartment by fibrosis [17] oedema [18] and amyloid [19]. From native and post-contrast T1, we can calculate extracellular volume fraction (ECV) which represents the interstitial volume [20]. Expansion of interstitial volume occurs with diffuse fibrosis, oedema and infiltrative diseases [21]. Recent studies have shown the improvement of these mapping techniques in the diagnostic accuracy of CMR in patients with suspected myocarditis [22–24]. T1 and T2 mapping sequences accurately diagnoses interstitial edema and extracellular space expansion and can potentially detect acute allograft rejection. In heart transplantation, few studies have assessed these mapping techniques [25–30]. Marie et al., more than 10 years ago, analysed T2 mapping in acute heart transplant rejection using a black-blood sequence on a low 0.5 Tesla magnet [25, 26]. They found that a T2 value ≥56 ms had an accurate detection of acute cardiac rejection defined by grade ≥ 2 (sensitivity 89%, specificity 70%). Bonnemains et al. confirmed these results on a higher field magnet (1.5 T) on 60 patients with a T2 value > 60 ms associated with risk of rejection higher than grade 2 [30].

To our knowledge, no studies have evaluated multisequential approach using T2 mapping, T1 mapping and ECV in heart transplant patients. The purpose of the study was to evaluate T2, T1 and ECV quantification as novel tissue markers to detect acute rejection in comparison to the gold standard strategy based on EMB.

## Methods

### Study population

The local committee approved this prospective study conducted from February 2012 to June 2015 in the Cardiology Department at the *Centre Hospitalier et Universistaire de Tours*, France. We recruited adult heart transplant patients receiving EMB within the first-year post transplantation. To maximize the number of CMR with rejection, most of the patients with suspected acute rejection during the study period performed CMR close to routine EMB and before acute rejection therapy. Patients were excluded if they had a contra-indication to CMR (e.g., pacemaker, defibrillator) or to gadolinium injection (serum creatinine ≥200 μmol/L). In addition, 34 local controls (age 54 ± 19 years) were included to determine the reference values of T2, T1 mapping and ECV at the mid left ventricular (LV) level.

### CMR acquisition

CMR examinations were performed using a 1.5 Tesla magnet (Magnetom Avanto, Siemens® Healthineers, Erlangen, Germany) with a 32-channel cardiac phased array coil.

LV and right ventricular (RV) systolic function was assessed using standard electrocardiogram (ECG) gated cine balanced steady state free precession (bSSFP) sequences acquired in the LV short-axis view using the following scan parameters: repetition time 37.3 ms, echotime 1.21 ms, flip angle 80°, slice thickness 6 mm, matrix 256 × 256, filed of view 320x320mm. LV and RV volumetric analyses were performed using Argus software (Siemens® Healthineers) to determine LV and RV ejection fraction, end-systolic volume, end-diastolic volume and myocardial mass. Standard method of LV functional analysis was performed by manually tracing endocardial and epicardial contours in the short-axis view.

Myocardial T2 mapping using a T2-prepared bSSFP sequence with 3 different T2 echo time using the following scan parameters: (echotime (ms): 0,24,55; image matrix 96 × 160, flip angle 40°, TR = 3xRR, total acquisition time of 7 heartbeats) was acquired through the LV at 3 levels (basal, mid and apical) with a respiratory and motion correction. T1-mapping sequence was based on the “modified Look-Locker inversion recovery” (MOLLI) sequence acquired through the LV at 3 levels (basal, mid and apical). Myocardial T1 mapping using a single shot bSSFP image was performed before gadolinium injection (0.2 mmol/kg gadoterique acid, Dotarem®, Guerbet, SA, Villepinte, France) and after 15 min. Acquisition schema 5(3)3 and 4(1)3(1)2 were used for pre and post contrast respectively. Other scan parameters included: echotime = 1.12 ms, acquisition matrix = 218 × 256, flip angle = 40°.

### Image analysis

Images were analyzed by a cardiologist blinded to the patient’s clinical data using certified specific software (Argus, Siemens, Medical solution).

**Fig. 1:**
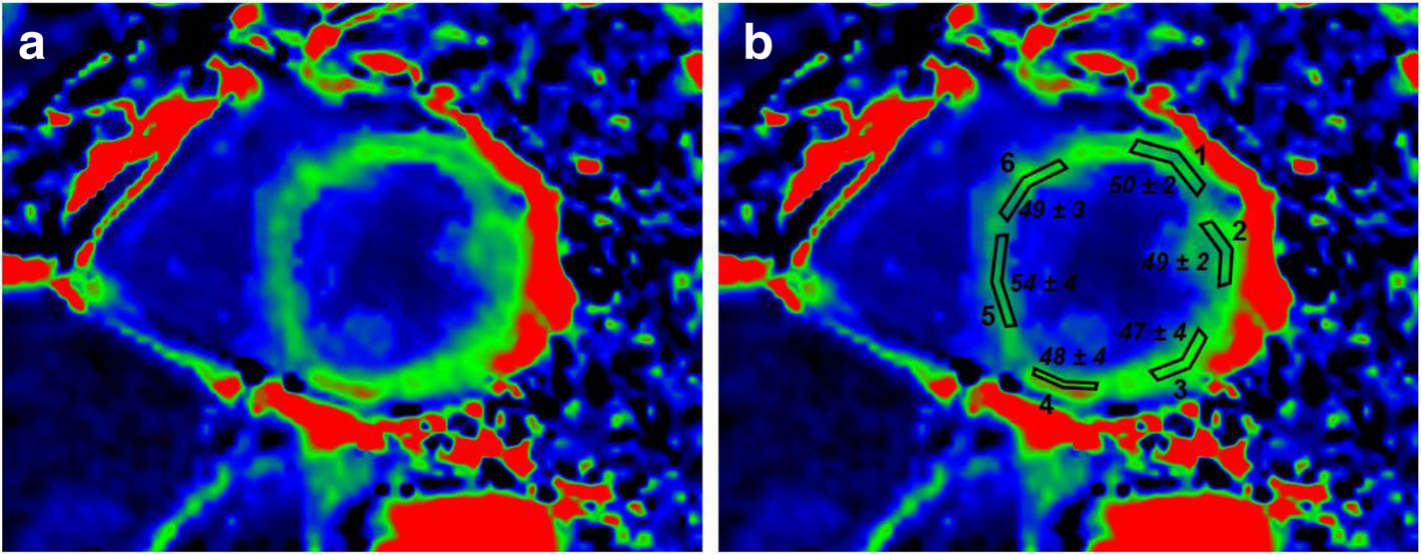
Mid left ventricle T2 mapping. T2 mapping in short axis at mid level without contouring (**a**) and with manual contouring (**b**) to define a region of interest (ROI) per segment

The short axis slice was segmented in 6 (at basal and mid-level) and in 4 (at apical level) equiangular segment model with the RV-LV junction as the reference point according to the American Heart Association 17 segments model [31]. Manual contouring was used to define a region of interest (ROI) per segment (Fig. 1).

Segmental T2 and T1 values were calculated using signal intensities from ROIs pixel by pixel. Global T2 and T1 values were obtained by adding values of each ROI dividing by the number of segments.

ECV was calculated using the following formula [20]:

$$ \mathbf{ECV}=\left(1-\mathrm{Haematocrit}\right)\ast \frac{\Delta \mathrm{R}1\ \mathrm{myocardium}}{\Delta \mathrm{R}1\ \mathrm{blood}} $$

$$ \boldsymbol{\Delta} \mathbf{R}\mathbf{1}\ \mathbf{myocard}=\frac{1}{\mathrm{T}1\ \mathrm{myocard}\ \mathrm{at}\ 15\ \mathrm{minutes}}-\frac{1}{\mathrm{T}1\ \mathrm{myocard}\ \mathrm{before}\ \mathrm{gadolinium}\ \mathrm{injection}} $$

$$ \boldsymbol{\Delta} \mathbf{R}\mathbf{1}\ \mathbf{blood}=\frac{1}{\mathrm{T}1\ \mathrm{blood}\ \mathrm{at}\ 15\ \mathrm{minutes}}-\frac{1}{\mathrm{T}1\ \mathrm{blood}\ \mathrm{before}\ \mathrm{gadolinium}\ \mathrm{injection}} $$

T1 relaxation of blood was obtained with a ROI placed in the middle of the LV cavity on T1 sequences before and after gadolinium injection for each of three level (basal, median and apical). Haematocrit was obtained from a blood test performed the day of the EMB.

### Endomyocardial biopsy

RV septal EMB was performed by experienced clinicians via jugular or femoral vein. At least four fragments were taken at each procedure. EMB was carried out either systematically according to the monitoring protocol of our center or in the case of clinical suspicion of rejection. The biopsies were interpreted by an experienced anatomopathologist, blinded to imaging data. The histological study of rejection was performed according to the 2004 International Society of Heart and Lung Transplantation (ISHLT) classification: grade 0R in the absence of rejection, grades 1R, 2R and 3R in light, moderate and severe rejection respectively [12]. The AMR was classified according to the 2011 ISHLT consensus: the biopsy was pAMR0 in the absence of rejection, pAMR1, pAMR2 or pAMR3 in the presence of specific histopathological lesions and/or in the case of positivity of immuno-histochemical analysis (CD68 or C4d positive) [32]. AMR was defined by a positive biopsy and/or circulating donor specific antibodies (DSA). Negative biopsy rejection was defined by the presence of graft dysfunction associated with circulating DSA.

### Statistical analysis

Quantitative variables were expressed as median with first and third interquartile range [Q1-Q3]. The distribution of qualitative variables was presented in numbers and percentages. The normality of the distribution was tested with the Kolmogorov-Smirnov test. First a non parametric test of Kruskal-Walis was used to test any difference between the three groups (control, no rejection and rejection). These global tests were followed by Dunn’s multiple comparisons test for pairwise comparison between the control group and no rejection (A) and between the control group and rejection group (B). The comparison of the quantitative variables for group *n* = 2 was carried out with the non-parametric Mann-Whitney test with a *p* <  0.05 value as the threshold of significance of the statistical test. To illustrate diagnostic performance, a Receiver-Operating Characteristic (ROC) analysis was performed to obtain areas under the curve (AUC) and their 95% confidence intervals (95% CI). The Youden index (sensitivity + specificity − 1) was used to establish the optimal cut-off values from the ROC curves. Diagnostic performance was also expressed as sensitivity, specificity, positive predictive value, negative predictive value and accuracy of the test. The accuracy of a diagnostic test is the ratio of correctly diagnosed patients (the sum of the “true positive” and the “true negative”) on the total number of patients. The inter and intra-observers agreement was assessed by the Bland and Altman plot expressed by the bias, standard deviation of bias et confidence interval at 95%. The Intraclass correlation coefficient and its 95% confidence interval were analysed by two way random test with absolute agreement using SPSS® Statistics V.25 (International Business Machines, Armonk, New York, USA).

Statistical analysiswere performed using GraphPad Prism® software (Version 6.0 for Windows - GraphPad Software Incorporation, La Jolla, California, USA).

## Results

### Study population

A total of 20 cardiac transplant patients were included providing 31 comparisons CMR-EMB. All EMBs were performed within 1 year of transplantation, all CMR were performed within 24 h of EMB. Table 1 summarizes the clinical, CMR and biopsy characteristics of the population. The median age of our population was 58 [49–64] years and the median LV ejection fraction was 63 [54–65] %. A total of 7 EMBs were positive for rejection including 6 cellular rejections and 1 AMR rejection. Cellular rejections ≥ grade 1R were specifically treated. All acute cellular rejections occurred within 6 months of transplantation.

**Table 1:** Baseline characteristics

Clinical data	*n* = 20
Age (years)	58 [49–64]
Male gender	14 (7%)
Body mass index (kg/m^2^)	26 [23–28]
Hypertension	10 (50%)
Diabetes	2 (10%)
Creatinin clearance (Cockcroft and Gault), ml/min	71 [52–94]
Age at transplant (years)	54 [44–60]
Donor age (years)	44 [31–50]
Ischemia time (minutes)	220 [202–233]
EMB	*n* = 31
*Negative (0R, pAMR0)*	24
*Positive with rejection*	7
Acute cellular rejection	6
Grade 1R	4
Grade 2R	2
Grade 3R	0
Antibody mediated rejection	1
pAMR1	1
pAMR2	0
*Time between transplant and EMB (days)*	80 [55–153]
CMR	*n* = 31
Time between transplant and CMR (days)	80 [56–156]
Time between BEM and CMR(days)	0 [0–1]

*EMB* endomyocardial biopsy, *CMR* cardiac magnetic resonance

Table 2 summarizes heart rate at the time of mapping acquisition and data on LV and RV parameters in the 2 groups (rejection and no rejection). No differences were significant.

**Table 2:** CMR analysis

	Rejection *n* = 7	No rejection *n* = 24	*p*
Heart rate (/min)	80 [70–93]	87 [82–94]	ns
Left Ventricle (LV)			
LV EDV(ml/m^2^)	64 [55–68]	67 [56–76]	ns
LV ESV(ml/m^2^)	25 [20–31]	22 [20–28]	ns
LV ejection fraction (%)	63 [54–67]	63 [54–65]	ns
Cardiac index (l/min/m^2^)	3.4 [2.8–4.0]	3.7 [2.8–4.0]	ns
LV mass (g/m^2^)	65 [56–72]	72 [62–89]	ns
Right ventricle (RV)			
RV EDV (ml/m^2^)	77 [56–79]	63[56–72]	ns
RV ESV (ml/m^2^)	27 [23–36]	27 [22–30]	ns
RV ejection fraction (%)	58 [55–64]	55 [53–63]	ns

*EDV* end-diastolic volume; *ESV end-systolic volume*

Table 3 summarizes CMR tissue characterization (T2, T1 and ECV) at mid level of the LV of the control group compared to rejection group and no rejection group.

**Table 3:** T2, T1 and ECV at mid level of the left ventricle in heart transplant patients and in controls

	Controls *n* = 34	Heart transplant patients		Kruskal-Wallis *p*-Value	Group A comparison	Group B comparison
		No rejection *n* = 24	Rejection *n* = 7			
T2 mapping (ms)	51.0 [49.0–53.0]	51.8 [50.1–53.6]	55.7 [54.0–59.7]	0.0131	ns	significant
T1 mapping before contrast (ms)	956 [948–971]	983 [956–1021]	993 [954–1050]	0.0178	significant	ns
T1 mapping after contrast (ms)	470 [448–501]	483 [461–500]	507 [485–538]	0.1705	ns	ns
ECV (%)	22.8 [21.6–24.5]	27.4 [24.4–31.5]	29.5 [27.8–36.3]	< 0.0001	significant	significant

ECV = Extra-Cellular Volume; ns = non significant.

Multiple group comparison was performed by the kruskal-wallis test expressed by the p-value. Comparison between each subgroup was tested with Dunn’s multiple comparison test; Group A comparison refers to a comparison between controls and the no rejection group, whereas Group B comparison refers to control and rejection group. Values are median [first (Q1) and third (Q3) quartiles].

### Global T2 mapping

**Fig. 2:**
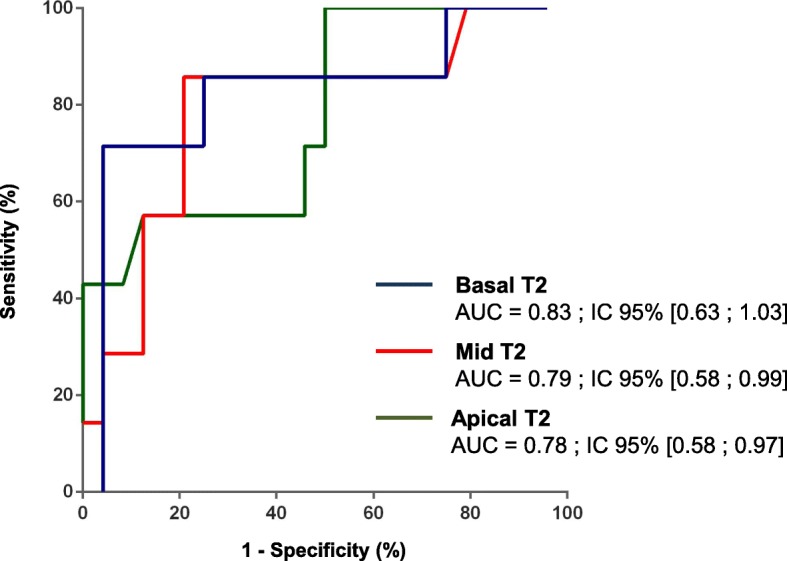
ROC curves of T2 mapping. ROC = receiver-operating characteristic; AUC = area under the curve; ECV = extracellular volume fraction

T2 values at mid level were significantly higher in patients with acute rejection compared to controls (Table 3) and compared to patients with no rejection at 3 levels (basal, mid and apical) (Table 4). The AUCs of the T2 mapping were statistically significant at 3 levels: basal: 0.83–95% CI [0.63–1.03]; mid: 0.79–95% CI [0.58–0.99] and apical: 0.78–95% CI [0.58–0.97]. Figure 2 shows the ROC curve of T2 mapping to identify acute rejection. A cut off of 57.7 ms at basal level had the best diagnostic accuracy (90%) with a sensitivity of 71%, a specificity of 96%, a positive predictive value of 83% and a negative predictive value of 92%. Image quality was excellent: no examination was discarded for inaccurate T2 measurement.

**Table 4:** T2 mapping, T1 mapping and ECV

	Rejection *n* = 7	No rejection *n* = 24	*p*
T2 mapping (ms)			
Global Basal	58.5 [55.0–60.3]	51.3 [49.5–55.2]	0.0074
Septal region	61.0 [54.0–62.0]	53.0 [49.0–57.0]	0,0148
Global Mid	55.7 [54.0–59.7]	51.8 [50.1–53.6]	0.0020
Septal region	58.0 [53.0–70.0]	53.0 [49.2–57.0]	ns
Apical	58.2 [54.0–63.7]	53.6[50.8–57.4]	0.0263
Septal region	57.0 [56.0–67.0]	54.0 [50.2–56.7]	ns
T1 mapping before contrast (ms)			
Basal	991 [952–1078]	990 [966–1042]	ns
Septal region	1013 [969–1039]	995 [952–1020]	ns
Mid	993 [954–1050]	983[956–1021]	ns
Septal region	984 [934–1063]	969[929–1010]	ns
Apical	1013 [980–1083]	1003 [957–1078]	ns
Septal region	1024 [962–1070]	1038 [963–1076]	ns
T1 mapping after contrast (ms)			
Basal	480 [432–506]	485 [453–504]	ns
Septal region	490 [459–511]	483 [444–498]	ns
Mid	507 [485–538]	483 [461–500]	ns
Septal region	480 [456–521]	473 [456–487]	ns
Apical	481[454–497]	452 [423–468]	ns
Septal region	489 [455–511]	455 [419–475]	ns
ECV (%)			
Basal	34.2 [32.8–37.4]	27.4 [24.6–30.6]	0,0061
Mid	29.5 [27.8–36.3]	27.4 [24.4–31.5]	ns
Apical	35.4 [30.2–39.0]	32.1 [28.5–36.9]	ns

*ECV* extra-cellular volume

T2 mapping intra observer and interobserver variabilities, analyzed on 19 patients by 2 experienced readers (EV and AD), were very good (Table 5).

**Table 5:** T2, T1 and T1 IV mapping. Intra and inter observer variabilities showed by Bland-Altman plot and intraclass correlation coefficient

		Bias	SD of Bias	95% confidence interval	Intraclass correlation	95% confidence interval
T2 mapping (msec)	Intra observer variability	0.7	1.7	[−2.6; +3.9]	0.983	[−0.957; 0.993]
	Inter observer variability	0.3	0.9	[−1.5; + 2.0]	0.949	[− 0.868; 0.980]
T1 mapping before contrast (msec)	Intra observer variability	−4	21	[−47; + 38]	0.995	[−0.989; 0.998]
	Inter observer variability	4	20	[−36; + 44]	0.944	[−0.862; 0.926]
T1 mapping after contrast (msec)	Intra observer variability	−6	7	[−20; + 9]	0.987	[−0.967 0.995]
	Inter observer variability	−1	5	[−12; + 9]	0.963	[−0.826; 0.988]

Intra observer and inter observer variabilities were evaluated by the Bland and Altman plot. Results are expressed as Bias, standard deviation of Bias and the 95% confident interval. Intraclass correlation coefficient and his 95% confidence interval were analyzed by two way random test with absolute agreement

### Segmental T2 mapping

T2 values were significantly higher in all segments in patients with acute rejection. A total of 489 segments out of 496 (98.6%) were properly analyzed.

### T1 mapping

Native T1 and post-contrast T1 values at 3 levels were not significantly different in patients with acute rejection compared to patients with no rejection (Table 4). Compared to healthy controls, only native T1 values were significantly higher (Table 3). Of the 31 CMRs imaging, pre-contrast T1 mapping values were obtained for 29 basal level examinations, 31 median level examinations and 29 apical level examinations. The T1 maps at 15 min after injection of the contrast agent were measured on the basal level myocardium for 27 examinations, at the median level for 28 examinations and at the apex for 27 examinations. Native and post contrast T1 reproducibilities were very good (Table 5).

### Extracellular volume

**Fig. 3:**
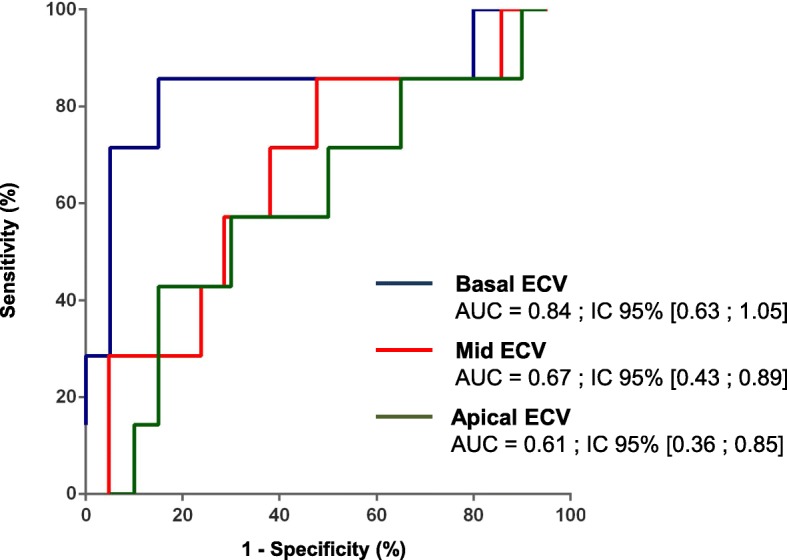
ROC curves of ECV. ROC = receiver-operating characteristic; AUC = area under the curve; ECV = extracellular volume fraction

ECV at mid level was significantly higher in heart transplant patients compared to healthy controls (Table 3). ECV at basal level was significantly higher in patients with acute rejection, not at mid and apical levels (Table 4). The AUC of ECV was statistically significant at basal level: 0.84–95% CI [0.63–1.05]. Figure 3 shows the ROC curves of ECV at 3 levels. To identify acute rejection, a cut off of 32% at basal level had the best accuracy (85%) with a sensitivity of 86%, a specificity of 85%, a positive predictive value of 67% and a negative predictive value of 94%. 4 ECV measurements were excluded on basal level, 3 on mid-level and 4 on apical level for inaccurate examination.

### Combined CMR parameters: T2 mapping and ECV

**Fig. 4:**
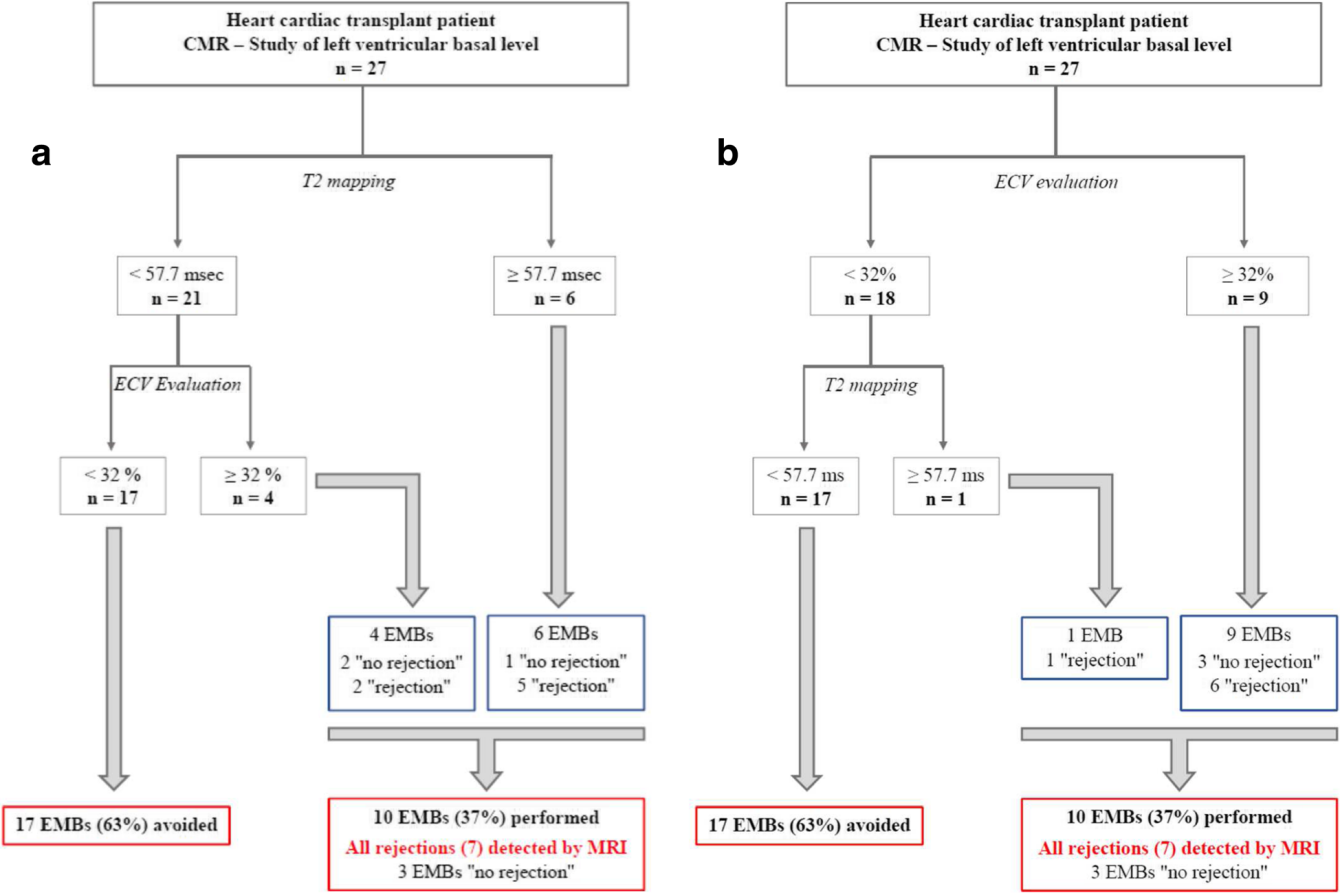
Schematic workflow applied in our population based on 2 pathways: T2 myocardial mapping supplemented by ECV measurement (**a**) and ECV supplemented by T2 mapping (**b**) on the basal level of the left ventricle. ECV = extracellular volume fraction

We have proposed a diagnostic strategy based on basal T2 first then basal ECV (Fig. 4a) and basal ECV first then T2 (Fig. 4b) in our population.

Cutoffs for T2 and ECV values are showed but may not be extrapolated to other centers due to a variability of these parameters with different magnet, vendors and field strengths. The threshold for T2 and ECV were those previously defined by the Youden index.

If we use basal T2 ≥ 57.7 ms as the first factor to diagnose acute rejection (Fig. 4a), 6 patients were positives; among them, 5 had an acute rejection confirmed on EMB (1 false positive case). When we added basal ECV ≥ 32%, 2 more patients were diagnosed with acute rejection confirmed on EMB.

If we use basal ECV ≥ 32% as the first factor (Fig. 4b), there were more false positives cases (3), but only 1 acute rejection confirmed on EMB was missed.

Native T2 diagnoses fewer false positive cases than ECV but misses more cases with the great advantage of the absence of contrast administration. When we combined T2 and ECV, sensibility and negative predictive value were each 100%. With that diagnostic strategy, CMR was able to detect all acute cardiac rejections and avoid 63% EMB (17 of 27). Only 3 BEMs would have been carried out without any rejection.

## Discussion

To our knowledge, this is the first study combining CMR T2 and T1 mapping with segmental and global analysis in a prospective cohort of heart transplant patients. The major findings were:

Basal T2 and basal ECV had a high diagnostic accuracy to identify patient with acute rejection. Combining T2 and ECV increased the sensitivity leading to the detection of all cardiac rejection episodes and potentially decreasing by 63% the number of EMBs. Segmental analysis of T2 demonstrated that acute rejection is a diffuse process involving all segments of the myocardium.

Because of interstitial edema and lymphocyte infiltrate, free water content is increased in acute rejection. As a result, tissue T2 relaxation time is increased and causes hyperintense areas on T2 weighted image in comparison with normal remote myocardium. Previous studies did not show significant increase in overall myocardial T2 signal ratios in patients with cellular rejection [33, 34]. More recently, Krieghoff et al. [35] in 73 heart transplant patients found that edema ratio had a 63% sensitivity, 78% specificity, 30% positive predictive value and 93% negative predictive value for cellular acute rejection grade ≥ 1b (ISHLT 1990 classification [36]).

Nevertheless, these sequences are limited by a low signal-to-noise ratio, motion artifacts, variable signal intensity and the possibility of inflammation of the skeletal muscle distorting the T2 ratio results. One of the strengths of T2 mapping is the possibility to measure T2 relaxation times in the absence of a ROI in the skeletal muscle and without comparison with normal myocardium. Current T2 mapping sequences appear robust and have led to improve image quality and reproducibility [37]. Our study confirms the high image quality of T2 mapping sequence with 98.6% of the analyzable segments (489/496) in accordance to Usman study (6 of 74 scans excluded) [29] and Butler study (6 of 77 scans excluded) [28].

Early studies in animal trials have showed, with histologic rejection, an increased T2 relaxation time with T2 values in rejecting animal from 46 to 68 ms [38, 39]. However, these results were limited by older sequence techniques on variable magnet (from 0.25 to 1.5 Tesla).

In humans, several studies have compared T2 relaxation times to transplant rejection [25–30]. Marie et al. [26], on 123 comparisons CMR-EMB using inversion-recovery/spin echo sequences on a low 0.5 T magnet, showed that a T2 ≥ 56 ms had sensitivity of 89% and specificity of 70% to detect moderate acute rejection using the previous ISHLT 1990 criteria [36]. More recently, Usman et al. [29] using updated imaging sequence (single shot T2 prepared bSSFP) and updated grading ISHLT classification, found that the optimal T2 time cut off was 56.4 ms with sensitivity of 86.5% and specificity of 94.6% to diagnose acute rejection in 8 rejection cases. Butler et al. [28], on 73 comparisons EMB-CMR using a modified half Fourier acquisition single shot turbo spin echo sequence, found that T2 relaxation time was 63 ± 6 ms in 15 patients with positive biopsy (defined by grade ≥ 2R or antibody mediated rejection) versus 57 ± 6 ms in 58 patients with negative biopsy. However, the authors did not use most recent T2 pre-bSSFP less sensitive to motion artifacts and incomplete blood suppression. A recent study [40] is not in agreement with the current study and the 3 other previously mentioned [26, 28, 29]. Miller et al. [40], on 88 comparisons CMR-EMB using a similar T2 prepared bSSFP sequence to ours, did not find significantly difference in T2 values between rejectors and non rejectors (58.8 ± 3.5 vs 57.0 ± 3.2, *p* = 0.242). However, the study design was different with statistical adjustment for serial measurements within the same patient. As discussed by the authors, if the statistical methods used by Usman [29] and Marie [26] were applied, the differences in T2 (and T1) between cardiac rejection group would be significant. In accordance with Usman et al. [29], we found that a T2 time cut-off 57.7 ms had sensitivity of 70%, specificity of 96% and an excellent diagnostic accuracy of 90% to diagnose acute rejection. However, our definition of significant acute cellular rejection was different from the 2 aforementioned studies [28, 29]. In our study, histological grade ≥ 1R was considered relevant rejection and treated (either changes in long term medication or IV steroids). In the other studies, only cellular rejection episodes ≥2R were relevant. A revision of the ISHLT-1990 criteria has been proposed merging the previous grade 1A, 1B and 2 within new 1R [12, 36]. The main difference from the previous set of criteria includes the addition of the ISHLT-1990 grade 2 (single focus of myocyte injury) with the mild 1R category. It has been demonstrated that grade 2 biopsies had significantly more edema (higher T2) than grade 1A and 1B [26]. Grade 1R has now become unspecific and 1R biopsies correspond to very different situations. A similar T2 cut off between our study and Usman’s can be explained by a majority of ISHLT-1990 grade 2 categories in our group of rejection. A large CMR multicenter study in heart transplant patients is ongoing to confirm diagnostic value of T2 mapping and to study the prediction value of T2 when biopsies are negative [41].

Until now, myocardial signal intensity was measured as ROI in the septum most of the time. Interestingly, we performed a segmental T2 analysis by measuring T2 myocardial signal intensity as ROI in the 17 American Heart Association segment models [31]. Although rejection’s distribution is often heterogeneous on biopsies, we have demonstrated that acute graft rejection is a diffuse process involving all segments of the myocardium. However, measuring T2 relaxation time in each segment is time consuming and does not provide a better diagnostic accuracy than the global T2 measurement. In daily life, we do not recommend performing a segmental T2 analysis. A global T2 measurement or a ROI in the septum corresponding to the anteroseptal and inferoseptal segments seem to be accurate.

In contrast to T2 studies, limited data are available on T1 mapping. Increased native T1 values have been shown in patients with myocarditis compared with controls [23, 24]. Although the mechanism is unclear, it could reflect an increase in intracellular and extracellular water content, possibly related to potential T2 effects in the T1 mapping sequence [42]. In heart transplant patients, data on T1 quantification are scare. Early studies in animal trials have found a prolonged T1 relaxation time correlated to the severity of rejection [39]. In humans, previous studies did not confirm these results [43].

Although our results cannot be compared to previous studies with different employed CMR sequences or different timing post transplantation [40], we confirmed that T1 mapping values before and after contrast injection were not significantly increased in the presence of rejection. Measuring absolute myocardial T1 values is limited due to several factors that can alter T1 measurement such as the acquisition scheme, magnetization transfer, flow and T2 effect [44, 45]. Moreover, T1 values vary with the CMR sequence and the algorithm of T1 calculation leading to discrepancies when comparing results between different studies. In contrast, ECV quantification should not be affected by confounding variables mentioned before. Values of myocardial ECV are very similar in different studies in the range of 24–28% [46, 47]. ECVs values also show better agreement with histological measure of the collagen volume fraction than isolated post contrast T1 [48].

Our data demonstrated that basal ECV had an excellent sensitivity of 89% and a good specificity of 77% to diagnose acute rejection. ECV at apical level was not significantly different in patient with acute rejection probably due to the poor image quality in apical segments and a thin wall.

When we use a multi-parametric sequential approach combining the excellent specificity of T2 (96%) with the good specificity of ECV (85%), we identified all acute rejection episodes potentially decreasing by 63% the need of EMB (Fig. 4). There has been little previous investigation on multi-sequential approach in heart transplant patients. Using T2 weighted imaging early and late gadolinium Enhancement (LGE), Taylor et al. [33], on 50 heart transplant recipients, found that the presence of either elevated early relative myocardial contrast enhancement (> 3.5) or increased relative STIR intensity (> 2) had sensitivity of 100% and specificity of 73% for confirmed acute rejection. More recently, using myocardial tissue characterization (T1 and T2 mapping and LGE) and regional ventricular function (mid ventricular short axis tagged images), Miller et al. [40], demonstrated significant reduction in circumferential strain without difference in T1 and T2 values.

### Study limitations

Our study is limited by its small sample size. We therefore were not able to compare patients with cellular rejection to patients with AMR. With only one patient with a control CMR 3 months after the initial episode, we were not able to assess CMR as a useful tool for the response to therapy. CMR was performed close to routine EMB but not always the day of the biopsy. However, CMR was always performed before acute rejection therapy. CMR was performed preferentially in patients who had a suspected rejection on the basis of results from clinical and echocardiography data which precludes the generalization of our findings. We do not think that this selection of patients might have had an important impact on our results.

## Conclusion

Our study suggests evidence for using a multi-parametric sequential approach to diagnose acute rejection in heart transplant recipients. Basal T2 mapping combining with basal ECV provided the best diagnostic accuracy leading to a potential reduction in the need for invasive EMBs by more than 50%. This is a preliminary study with a small sample size. Larger studies are required to confirm these data.

## Abbreviations

AMR: Antibody-mediated rejection
AUC: Area under the curve
bSSFP: Balanced steady state free precession
CMR: Cardiovascular magnetic resonance
DSA: Donor specific antibodies
ECG: Electrocardiogram
ECV: Extracellular volume fraction
EMB: Endomyocardial biopsy
ISHLT: International Society of Heart and Lung Transplantation
LGE: Late gadolinium enhancement
LV: Left ventricle/left ventricular
MOLLI: Modified Look Locker inversion recovery
ROC: Receiver-operating characteristic
ROI: Region of interest
RV: Right ventricle/right ventricular

## References

[1] Chambers DC, Yusen RD, Cherikh WS, Goldfarb SB, Kucheryavaya AY, Khusch K, et al. The Registry of the International Society for Heart and Lung Transplantation: Thirty-fourth Adult Lung And Heart-Lung Transplantation Report-2017; Focus Theme: Allograft ischemic time. J Heart Lung Transplant Off Publ Int Soc Heart Transplant. 2017;36:1047–59. 10.1016/j.healun.2017.07.016 28784324

[2] Kofler S, Bigdeli AK, Kaczmarek I, Kellerer D, Müller T, Schmoeckel M (2009). Long-term outcomes after 1000 heart transplantations in six different eras of innovation in a single center. Transpl Int Off J Eur Soc Organ Transplant.

[3] Patel JK, Kobashigawa JA (2006). Should we be doing routine biopsy after heart transplantation in a new era of anti-rejection?. Curr Opin Cardiol.

[4] Yun KL, Niczyporuk MA, Daughters GT, Ingels NB, Stinson EB, Alderman EL (1991). Alterations in left ventricular diastolic twist mechanics during acute human cardiac allograft rejection. Circulation.

[5] Costanzo MR, Dipchand A, Starling R, Anderson A, Chan M, Desai S (2010). The International Society of Heart and Lung Transplantation Guidelines for the care of heart transplant recipients. J Heart Lung Transplant Off Publ Int Soc Heart Transplant.

[6] Saraiva F, Matos V, Gonçalves L, Antunes M, Providência LA (2011). Complications of Endomyocardial Biopsy in Heart Transplant Patients: A Retrospective Study of 2117 Consecutive Procedures. Transplant Proc.

[7] Baraldi-Junkins C, Levin HR, Kasper EK, Rayburn BK, Herskowitz A, Baughman KL (1993). Complications of endomyocardial biopsy in heart transplant patients. J Heart Lung Transplant Off Publ Int Soc Heart Transplant.

[8] Marelli D, Esmailian F, Wong SY, Kobashigawa JA, Kwon MH, Beygui RE (2009). Tricuspid valve regurgitation after heart transplantation. J Thorac Cardiovasc Surg.

[9] Spiegelhalter DJ, Stovin PG (1983). An analysis of repeated biopsies following cardiac transplantation. Stat Med.

[10] Fishbein MC, Kobashigawa J (2004). Biopsy-negative cardiac transplant rejection: etiology, diagnosis, and therapy. Curr Opin Cardiol.

[11] Nielsen H, Sørensen FB, Nielsen B, Bagger JP, Thayssen P, Baandrup U. Reproducibility of the acute rejection diagnosis in human cardiac allografts. The Stanford Classification and the International Grading System. J Heart Lung Transplant Off Publ Int Soc Heart Transplant. 1993;12:239–43. 8476896

[12] Stewart S, Winters GL, Fishbein MC, Tazelaar HD, Kobashigawa J, Abrams J (2005). Revision of the 1990 working formulation for the standardization of nomenclature in the diagnosis of heart rejection. J Heart Lung Transplant Off Publ Int Soc Heart Transplant.

[13] Bellenger NG, Burgess MI, Ray SG, Lahiri A, Coats AJ, Cleland JG (2000). Comparison of left ventricular ejection fraction and volumes in heart failure by echocardiography, radionuclide ventriculography and cardiovascular magnetic resonance; are they interchangeable?. Eur Heart J.

[14] Eitel I, Friedrich MG (2011). T2-weighted cardiovascular magnetic resonance in acute cardiac disease. J Cardiovasc Magn Reson Off J Soc Cardiovasc Magn Reson.

[15] Verhaert D, Thavendiranathan P, Giri S, Mihai G, Rajagopalan S, Simonetti OP (2011). Direct T2 quantification of myocardial edema in acute ischemic injury. JACC Cardiovasc Imaging.

[16] Friedrich MG, Sechtem U, Schulz-Menger J, Holmvang G, Alakija P, Cooper LT (2009). Cardiovascular Magnetic Resonance in Myocarditis: A JACC White Paper. J Am Coll Cardiol.

[17] Nakamori S, Dohi K, Ishida M, Goto Y, Imanaka-Yoshida K, Omori T, et al. Native T1 Mapping and Extracellular Volume Mapping for the Assessment of Diffuse Myocardial Fibrosis in Dilated Cardiomyopathy. JACC Cardiovasc Imaging. 2017;11:48–59. 10.1016/j.jcmg.2017.04.006 28624408

[18] Goldfarb JW, Arnold S, Han J (2007). Recent myocardial infarction: assessment with unenhanced T1-weighted MR imaging. Radiology.

[19] Fontana M, Banypersad SM, Treibel TA, Maestrini V, Sado DM, White SK (2014). Native T1 mapping in transthyretin amyloidosis. JACC Cardiovasc Imaging.

[20] Flett AS, Hayward MP, Ashworth MT, Hansen MS, Taylor AM, Elliott PM (2010). Equilibrium contrast cardiovascular magnetic resonance for the measurement of diffuse myocardial fibrosis: preliminary validation in humans. Circulation.

[21] Ugander M, Oki AJ, Hsu L-Y, Kellman P, Greiser A, Aletras AH (2012). Extracellular volume imaging by magnetic resonance imaging provides insights into overt and sub-clinical myocardial pathology. Eur Heart J.

[22] Thavendiranathan P, Walls M, Giri S, Verhaert D, Rajagopalan S, Moore S (2012). Improved detection of myocardial involvement in acute inflammatory cardiomyopathies using T2 mapping. Circ Cardiovasc Imaging.

[23] Ferreira VM, Piechnik SK, Dall’Armellina E, Karamitsos TD, Francis JM, Ntusi N (2014). Native T1-mapping detects the location, extent and patterns of acute myocarditis without the need for gadolinium contrast agents. J Cardiovasc Magn Reson Off J Soc Cardiovasc Magn Reson.

[24] Radunski UK, Lund GK, Stehning C, Schnackenburg B, Bohnen S, Adam G (2014). CMR in patients with severe myocarditis: diagnostic value of quantitative tissue markers including extracellular volume imaging. JACC Cardiovasc Imaging.

[25] Marie PY, Carteaux JP, Angioï M, Marwan NS, Tzvetanov K, Escanye JM (1998). Detection and prediction of acute heart transplant rejection: preliminary results on the clinical use of a “black blood” magnetic resonance imaging sequence. Transplant Proc.

[26] Marie PY, Angioï M, Carteaux JP, Escanye JM, Mattei S, Tzvetanov K (2001). Detection and prediction of acute heart transplant rejection with the myocardial T2 determination provided by a black-blood magnetic resonance imaging sequence. J Am Coll Cardiol.

[27] Aherne T, Yee ES, Tscholakoff D, Gollin G, Higgins C, Ebert PA (1988). Diagnosis of acute and chronic cardiac rejection by magnetic resonance imaging: a non-invasive in-vivo study. J Cardiovasc Surg (Torino).

[28] Butler CR, Savu A, Bakal JA, Toma M, Thompson R, Chow K (2015). Correlation of cardiovascular magnetic resonance imaging findings and endomyocardial biopsy results in patients undergoing screening for heart transplant rejection. J Heart Lung Transplant Off Publ Int Soc Heart Transplant.

[29] Usman AA, Taimen K, Wasielewski M, McDonald J, Shah S, Giri S (2012). Cardiac Magnetic Resonance T2 Mapping in the Monitoring and Follow-up of Acute Cardiac Transplant Rejection: A Pilot Study. Circ Cardiovasc Imaging.

[30] Bonnemains L, Villemin T, Escanye J-M, Hossu G, Odille F, Vanhuyse F (2014). Diagnostic and prognostic value of MRI T2 quantification in heart transplant patients. Transpl Int.

[31] Cerqueira MD, Weissman NJ, Dilsizian V, Jacobs AK, Kaul S, Laskey WK (2002). Standardized myocardial segmentation and nomenclature for tomographic imaging of the heart. A statement for healthcare professionals from the Cardiac Imaging Committee of the Council on Clinical Cardiology of the American Heart Association. J Nucl Cardiol Off Publ Am Soc Nucl Cardiol.

[32] Kobashigawa J, Crespo-Leiro MG, Ensminger SM, Reichenspurner H, Angelini A, Berry G (2011). Report from a consensus conference on antibody-mediated rejection in heart transplantation. J Heart Lung Transplant Off Publ Int Soc Heart Transplant.

[33] Taylor AJ, Vaddadi G, Pfluger H, Butler M, Bergin P, Leet A (2010). Diagnostic performance of multisequential cardiac magnetic resonance imaging in acute cardiac allograft rejection. Eur J Heart Fail.

[34] Almenar L, Igual B, Martínez-Dolz L, Arnau MA, Osa A, Rueda J (2003). Utility of cardiac magnetic resonance imaging for the diagnosis of heart transplant rejection. Transplant Proc.

[35] Krieghoff C, Barten MJ, Hildebrand L, Grothoff M, Lehmkuhl L, Lücke C (2014). Assessment of sub-clinical acute cellular rejection after heart transplantation: comparison of cardiac magnetic resonance imaging and endomyocardial biopsy. Eur Radiol.

[36] Billingham ME, Cary NR, Hammond ME, Kemnitz J, Marboe C, McCallister HA (1990). A working formulation for the standardization of nomenclature in the diagnosis of heart and lung rejection: Heart Rejection Study Group. The International Society for Heart Transplantation. J Heart Transplant.

[37] Giri S, Chung Y-C, Merchant A, Mihai G, Rajagopalan S, Raman SV (2009). T2 quantification for improved detection of myocardial edema. J Cardiovasc Magn Reson.

[38] Tscholakoff D, Aherne T, Yee ES, Derugin N, Higgins CB (1985). Cardiac transplantations in dogs: evaluation with MR. Radiology.

[39] Nishimura T, Sada M, Sasaki H, Yutani C, Kozuka T, Amemiya H (1987). Identification of cardiac rejection with magnetic resonance imaging in heterotopic heart transplantation model. Heart Vessels.

[40] Miller CA, Naish JH, Shaw SM, Yonan N, Williams SG, Clark D (2014). Multiparametric cardiovascular magnetic resonance surveillance of acute cardiac allograft rejection and characterisation of transplantation-associated myocardial injury: a pilot study. J Cardiovasc Magn Reson.

[41] Bonnemains L, Cherifi A, Girerd N, Odille F, Felblinger J (2015). Design of the DRAGET Study: a multicentre controlled diagnostic study to assess the detection of acute rejection in patients with heart transplant by means of T2 quantification with MRI in comparison to myocardial biopsies. BMJ Open.

[42] Chow K, Flewitt J, Pagano JJ, Green JD, Friedrich MG, Thompson RB (2012). T2-dependent errors in MOLLI T1 values: simulations, phantoms, and in-vivo studies. J Cardiovasc Magn Reson.

[43] Kurland RJ, West J, Kelley S, Shoop JD, Harris R, Carr EA (1989). Magnetic resonance imaging to detect heart transplant rejection: sensitivity and specificity. Transplant Proc.

[44] Moon JC, Messroghli DR, Kellman P, Piechnik SK, Robson MD, Ugander M (2013). Myocardial T1 mapping and extracellular volume quantification: a Society for Cardiovascular Magnetic Resonance (SCMR) and CMR Working Group of the European Society of Cardiology consensus statement. J Cardiovasc Magn Reson Off J Soc Cardiovasc Magn Reson.

[45] Piechnik SK, Ferreira VM, Dall’Armellina E, Cochlin LE, Greiser A, Neubauer S (2010). Shortened Modified Look-Locker Inversion recovery (ShMOLLI) for clinical myocardial T1-mapping at 1.5 and 3 T within a 9 heartbeat breathhold. J Cardiovasc Magn Reson Off J Soc Cardiovasc Magn Reson.

[46] Lee JJ, Liu S, Nacif MS, Ugander M, Han J, Kawel N (2011). Myocardial T1 and extracellular volume fraction mapping at 3 T. J Cardiovasc Magn Reson.

[47] Rogers T, Dabir D, Mahmoud I, Voigt T, Schaeffter T, Nagel E (2013). Standardization of T1 measurements with MOLLI in differentiation between health and disease--the ConSept study. J Cardiovasc Magn Reson Off J Soc Cardiovasc Magn Reson.

[48] Sibley CT, Noureldin RA, Gai N, Nacif MS, Liu S, Turkbey EB (2012). T1 Mapping in cardiomyopathy at cardiac MR: comparison with endomyocardial biopsy. Radiology.

